# Mental Health and Perceived Access to Care among People Who Inject Drugs in Athens, Greece

**DOI:** 10.3390/jcm10061181

**Published:** 2021-03-12

**Authors:** Despina Pampaka, Katerina Pantavou, George Giallouros, Eirini Pavlitina, Leslie D. Williams, Daniele Piovani, Stefanos Bonovas, Georgios K. Nikolopoulos

**Affiliations:** 1 Cyprus International Institute for Environmental and Public Health, Cyprus University of Technology, Limassol 3041, Cyprus; despina.pampaka@cut.ac.cy; 2 Medical School, University of Cyprus, Nicosia 2029, Cyprus; pantavou.katerina@ucy.ac.cy (K.P.); giallouros.giorgos@ucy.ac.cy (G.G.); 3 Department of Business and Public Administration, University of Cyprus, Nicosia 1678, Cyprus; 4 Transmission Reduction Intervention Project, Athens Site, 11527 Athens, Greece; pavlitina.eirini@gmail.com; 5 Division of Community Health Sciences, University of Illinois at Chicago School of Public Health, Chicago, IL 60612, USA; lesliedw@uic.edu; 6 National Development and Research Institutes, New York, NY 10010, USA; 7 Department of Biomedical Sciences, Humanitas University, 20090 Milan, Italy; dpiovani@hotmail.com (D.P.); sbonovas@gmail.com (S.B.); 8 IRCCS Humanitas Research Hospital, 20089 Milan, Italy

**Keywords:** PWID, HIV, social networks, mental health, medical care, recent infection

## Abstract

Poor mental health among human immunodeficiency virus (HIV)-positive people who inject drugs (PWID) may contribute to stigma, and together they act as barriers to medical care. This analysis aims to examine factors associated with the mental health of PWID and their network contacts, and the association of poor mental health with the experience of HIV-related stigmatizing events, with HIV-related social support, and with perceived access to care. Data were collected during the Transmission Reduction Intervention Project (TRIP) conducted in Athens, Greece (2013–2015). PWID (*n* = 292; *n* = 122 HIV-positive) were interviewed both at baseline and follow-up. Items of depression, anxiety, and general positive affect subscales of the Mental Health Inventory were used to explore the psychological distress and well-being of participants at follow-up. Items of the Access to Care Scale were used to evaluate perceived access to medical care at baseline and follow-up. Linear regression showed that unemployment was positively related to depression (β = 1.49, *p* = 0.019), while injecting drug use was a risk factor for a low general positive affect score (β = −3.21, *p* = 0.015). Poor mental health was not linked to HIV-related stigma or social support. Positive perception of access to care was associated in multivariable analyses with low depression (β = −0.22, *p* = 0.049). The perceived access to care score improved from baseline to follow-up (*p* = 0.019) and HIV-positive participants had a higher score than HIV-negative participants. Future interventions should include targets to improve the mental well-being of participants, reduce psychosocial distress, and minimize perceived barriers to accessing medical care.

## 1. Introduction

People who inject drugs (PWID) are vulnerable to mental health disorders [1], often experiencing the distressing circumstances of unemployment, unstable housing, and traumatic negative attitudes or discrimination (stigma). Mental health disorders increase the risk of human immunodeficiency virus (HIV) acquisition [2,3], while people living with HIV (PLHIV) experience heightened rates of mental health disorders, such as depression, dysthymia, and anxiety [2,4]. Forms of stress contributing to mental health disorders in HIV-infected individuals are stigma and discrimination, loss of social support resulting in isolation, potential loss of employment and relationships, changes in physical appearance or abilities, and stress related to getting medical care services [5]. Newly diagnosed HIV-positive people have a high risk of developing depression as the HIV diagnosis itself may trigger depressive symptoms [6,7]. The onset of depression and the latency period between diagnosis of HIV infection and diagnosis of mental health disorders may vary with gender [8]. Poor mental health decreases quality of life and negatively affects HIV disease progression [9]. Together with stigma, it has a negative impact on seeking care, adherence to antiretroviral treatment (ART), and clinic attendance [6,10,11,12,13,14,15]. Non-access to medical care is a significant obstacle in the global efforts to suppress the HIV epidemic through ART [16,17]. 

In times of economic recession, common mental health disorders tend to increase both among the general population and specific sub-populations, such as the unemployed, people in debt or facing financial difficulties, those with pre-existing mental health problems, and families with children [18]. Austerity cuts may include a decreased number of health infrastructures, a reduced capacity for health care facilities and services, reductions in the workforce and budget for medicines, as well as personal difficulty in responding to the healthcare costs that reduce accessibility to health care [19]. Economic recession can also result in epidemics of HIV due to, among other causes, increase in the size of risk networks or increasing rates of unsafe drug injection-related or sexual behaviors [20]. Exploring common mental health issues among PWID, and especially those who live with HIV, is particularly important in periods of economic and social crisis. Adequate support and suitable intervention programs could be necessary to improve mental health and access to care, and to reduce the stigma and risk of HIV transmission. 

Greece was severely affected by an economic crisis that started in 2008 causing intercommunal violence, population displacement, social movements, and cuts in governmental expenditures [20,21]. The general recession and austerity measures likely affected vulnerable population groups such as PWID. An increase in unemployment, a lack of (or reduced) support from families, and unstable housing resulted in changes in PWID behaviors and in the size and structure of their social networks. In 2011, the number of HIV-positive PWID increased rapidly from 10 to 20 per year before 2011 to more than 250, representing a 16-fold rise compared with 2010 [20,21]. As a response to this outbreak, among other measures, the Transmission Reduction Intervention Project (TRIP) was launched in Athens [22]. TRIP was a social network-based intervention, which was aimed at reducing the risk of HIV transmission by identifying recently infected PWID and linking them to care [22,23,24]. TRIP also traced PWID’s contacts in their social network in order to inform them about the high risk of infection in their social circles and to help them get tested [22]. 

This analysis investigated the mental health and perceived access to care of PWID and their social networks in Athens, Greece, in the context of a network-based intervention following a large HIV outbreak. Thus, it focused on (a) exploring mental health indicators in PWID, (b) examining whether mental health indicators were associated with HIV-related stigma or support experiences, and (c) testing whether mental health, HIV-related stigma, and HIV-related support affected perceived access to medical care.

## 2. Materials and Methods

### 2.1. Study Design and Setting

TRIP was conducted in Athens, Greece, between June 2013 and July 2015. Recruitment started with PWID infected with HIV either in the previous six months (recent seeds, RS) or longer than six months (control seeds with long-term HIV infection, LCS), who were referred by an allied prevention project (i.e., ARISTOTLE) [25] or by collaborating facilities. Seeds (RS and LCS) were asked to provide the names of, and help recruit, members of their social networks. Network members were people with whom seeds reported to be having sexual intercourse and/or injecting drugs with, as well as people from venues where the seeds met regularly for injecting drugs or having sexual intercourse. These contacts were traced in order to ask them to participate in TRIP and to inform them about the high risk of infection in their social network. Moreover, TRIP recruited HIV-negative people as controls (negative controls, NC). Eligible participants were individuals aged 18 years or older and able to be interviewed by the experienced TRIP personnel. Additional information about TRIP is described in Nikolopoulos et al. [22]. 

The interviews were conducted using a questionnaire and were carried out at both baseline and follow-up (at six months after enrollment). The questionnaire used at baseline included items to provide information on socio-demographics, injecting drug use, risk of drug injection-related and sexual behaviors, pre-exposure prophylaxis, access to medical care, drug use treatment, and stigmatizing experiences. It was also used to trace the participants’ network members who were at risk. A follow-up interview was scheduled six months after the baseline visit and the participants were requested to answer an extended version of the baseline questionnaire. The questionnaire at follow-up included additional items related to participants’ mental health (depression, anxiety, and general positive affect) and to the social support they had received over the past months. 

All TRIP participants were tested for HIV, and for recent HIV infection, with limiting antigen avidity (LAg) assay (Sedia^TM^ Biosciences Corporation; Beaverton, OR, USA), and for viral RNA, if they were HIV-infected. A median score of 1.5 or less in the standardized optical density (ODn) indicated an infection within 130 days (recent HIV infection). Participants who were HIV-negative at enrollment were examined again at follow-up to detect possible seroconversion. HIV-diagnosed participants were linked to care and treatment [24].

Ethical approval was provided by the Institutional Review Boards of the National Development and Research Institutes (NDRI) in New York and from the Hellenic Scientific Society for the Study of AIDS and Sexually Transmitted Diseases in Athens. All procedures performed were in accordance with the ethical standards of the institutional and/or national research committee and with the 1964 Helsinki Declaration and its later amendments or comparable ethical standards. Informed consent was obtained from all individuals who participated in TRIP.

### 2.2. Participants

**Figure 1:**
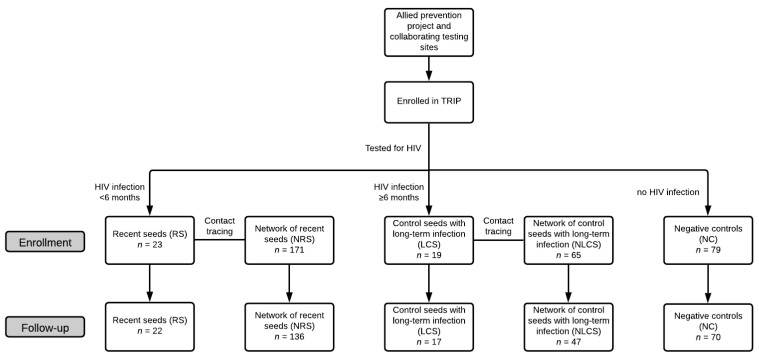
Flowchart of participant selection in Transmission Reduction Intervention Project (TRIP).

A total of 357 individuals enrolled at the baseline visit to TRIP. This was reduced to 292 at the follow-up visit due to loss to follow-up or unwillingness to participate, especially among the networks of RS and LCS (Figure 1). The participants were classified into groups based on: (a) the arm of the study, i.e., recent seeds (RS; *n* = 22, 7.5%), network members of recent seeds (NRS; *n* = 136, 46.6%), control seeds with long-term infection (LCS; *n* = 17, 5.8%), network members of control seeds with long-term infection (NLCS; *n* = 47, 16.1%), and HIV-negative controls (NC; *n* = 70, 24%); and (b) HIV status, i.e., HIV-positive (*n* = 122, 41.8%) and HIV-negative (*n* = 170, 58.2%). 

### 2.3. Questionnaire and Measures

Mental health, including psychological distress and well-being, were evaluated using depression, anxiety, and general positive affect subscales of the Rand Mental Health Inventory (MHI-38) [26]. This is a self-reporting tool with 38 items, used in population surveys, and does not form part of a clinical assessment for mental health disorders. The questionnaire included four items on depression, nine on anxiety, and ten on general positive affect (Supplement File, Table S1). Each item takes a score from 1 to 6, except for one item of the depression subscale that takes a score from 1 to 5. Therefore, the total score of depression subscale ranges between 4 and 23, of anxiety between 9 and 54, and of general positive affect between 10 and 60. High subscale scores indicate high depression, anxiety, and general positive affect.

Participants were asked to report four kinds of stigmatizing events they had experienced caused by people who thought they were recently infected with HIV (i.e., nasty comments, threats or attacks, denial of access to goods or forbiddance to go somewhere, and exclusion from social gatherings) [27]. Moreover, they were asked to report if they had received social support offered in three ways: (a) emotional, (b) concrete assistance, such as money or food, and (c) information about HIV-patient services, testing, or consultation. All stigma and social support items responses were in the format 1-*Yes* or 0-*No*. The total scores for HIV-related stigma and social support were calculated, and ranged from 0 to 4 and from 0 to 3, respectively. High scores indicated a high level of stigma or support. 

Perception of access to medical care was examined using five items from the Access to Care Scale [28]: (a) I am able to get medical care whenever I need it, (b) It is hard for me to get medical care in an emergency, (c) Sometimes I go without the medical care I need because it is too expensive, (d) I have easy access to the medical specialists that I need, and (e) Places where I can get medical care are very conveniently located. A five-point (0–4) Likert scale (0, *strongly disagree*; 4, *strongly agree*) was utilized to respond. The scores of items (b) and (c) were reversed so that high scores would indicate positive perception of access to care. A total score between 0 and 20 was obtained by adding the scores of (a) to (e). High scores indicated a positive perception of access to medical care.

### 2.4. Statistical Analysis

Chi-squared tests and effect sizes (Cramérs’ V), one-way analysis of variance (ANOVA), and independent sample *t*-tests were used to compare the socio-demographic characteristics of participants to the scores on depression, anxiety, and general positive affect. Comparisons were also conducted between HIV-positive and HIV-negative participants. The perceived access to care score between baseline and follow-up was compared using a *t*-test. Univariable linear regression models were used to explore potential risk factors for psychological distress (depression and anxiety) and well-being (general positive affect), and whether mental health affects HIV-related stigma and social support. Similarly, univariable and multivariable linear regression models were used to identify the correlates of perceived access to care scores and to investigate if perceived access to care scores could be predicted by HIV-related stigmatizing experiences, HIV-related support, and mental health, after controlling for socio-demographic characteristics. Independent variables found to be statistically significant at the univariable stage entered the multivariable selection process. The stigmatizing and social support experiences of TRIP participants were examined and described in detail in the study of Williams et al. [27].

All statistical analyses were conducted using STATA software (StataCorp. 2011, College Station, TX, USA) and statistical significance was defined at a *p*-value <0.05 using two-sided tests.

## 3. Results

### 3.1. Characteristics of Participants

The participants of TRIP at follow-up (*n* = 292) were mainly males (*n* = 231, 79.1%), of Greek nationality (*n* = 269, 92.1%), non-homeless (*n* = 252, 86.6%), with education up to high school (*n* = 250, 85.6%), and unemployed or unable to work (*n* = 232, 79.5%). About 53.4% (*n* = 156) of the participants were on drug/alcohol treatment at the time of their enrollment in TRIP. Participants’ characteristics were similar between the five arms of the study (Table 1). Differences were observed in the percentage of homelessness and drug injection use. HIV-negative controls had the lowest levels of homeless people (*n* = 2, 2.9%; *p* = 0.03; Cramér’s V = 0.19) and PWID (*n* = 35, 50%; *p* < 0.001; Cramér’s V = 0.28). Considering HIV status (Table 2), HIV-positive participants were more likely than HIV-negative participants to be homeless (19%, *n* = 23 versus 9.4%, *n* = 16; *p* = 0.02; Cramér’s V = 0.14), unemployed (86.9%, *n* = 106 versus 74.1%, *n* = 126; *p* = 0.008; Cramér’s V = 0.16), and PWID (88.5%, *n* = 108 versus 60.6%, *n* = 103; *p* < 0.001; Cramér’s V = 0.31).

### 3.2. Mental Health

Depression, anxiety, and general positive affect maximum scores of participants were 23, 52, and 60, respectively. Table 3 presents the mean scores of mental health subscales. Depression and anxiety mean scores were 14.1 and 32.5, respectively, while the mean score of general positive affect was 28.6. There was no significant difference in depression, anxiety, or general positive affect mean scores among the study arms or between HIV-positive and HIV-negative participants (*p* > 0.05). 

Univariable analyses (Table 4) revealed that the depression score was higher among those who were unemployed or unable to work compared to employed individuals (β = 1.49, *p* = 0.019). Moreover, the general positive affect score was lower among those who injected drugs over the past 6 months (β = −3.21, *p* = 0.015) than those who did not inject drugs. Other characteristics of the participants were not significantly related to mental health scores (*p* > 0.05). 

### 3.3. HIV-Related (for Recent Infection) Stigma and Social Support 

Stigma scores of the participants ranged between 0 and 4 (mean ± standard deviatio*n* = 0.3 ± 0.8). The most frequently reported HIV-related (for recent infection) stigma experience was receiving nasty comments (*n* = 49, 16.8%). Social support scores ranged between 0 and 3 while the mean was 0.4 (±0.9). The most common type of HIV-related support was offering information about where someone could get an HIV service/testing/consultation, etc. (*n* = 52, 17.8%). 

Univariable linear regression models showed that depression, anxiety, and general positive affect scores were not associated with the scores of HIV-related stigma or social support experiences.

### 3.4. Perceived Access to Care

The mean score of perceived access to care improved from baseline (9.7 ± 5.6) to follow-up (10.6 ± 5.4; *p* = 0.019). The most commonly reported barriers to care at follow-up were the cost of medical care (*strongly agree* *n* = 120, 41.1%) and the difficulty of accessing care in an emergency (*strongly agree* *n* = 116, 39.7%). 

Univariable linear regression showed that younger participants (β = −0.08, *p* = 0.045) or those with HIV (β = 1.49, *p* = 0.025) reported higher scores of perceived access to care than older or HIV-negative participants (Table 5).

Depression (β = −0.29, *p* < 0.001) and anxiety (β = −0.14, *p* < 0.001) were negatively associated with perceived access to care (Table 5). The opposite was found for general positive affect (β = 0.08, *p* = 0.022) and perceived access to care scores at baseline (β = 0.34, *p* < 0.001) that were positively associated with perceived access to care score at follow-up. Multivariable linear regression used to assess the simultaneous effect of all the statistically significant variables (*p* < 0.05) in the univariable analysis showed that only the HIV status (β = 1.32, *p* = 0.033) and the baseline perceived access to care score (β = 0.33, *p* < 0.001) were significantly correlated to perceived access to care at follow-up, while a weak association was found in the case of depression score (β = −0.22, *p* = 0.049) (Table 5). 

## 4. Discussion

Mental health in terms of psychological distress (depression and anxiety) and well-being (general positive affect) was examined in drug injecting networks in Athens, Greece, during a serious economic crisis. The impact of depression, anxiety, and general positive affect scores on HIV-related stigma, social support, and perceived access to care was investigated, and potential factors affecting mental health and perceived access to care were identified. Unemployment was a risk factor for depression, while HIV status and depression were related to perceived access to care. 

Previous studies have shown that depression and anxiety are more common among HIV-positive individuals [29,30,31,32]. However, in this study, the levels of psychological distress did not vary with HIV status. Similarly, there were no differences between the study arms, indicating that those who have been infected with HIV recently and those with a longer-term HIV infection had similar levels of depression and anxiety. Unemployment is a known risk factor for depression among HIV-positive individuals [33,34]. In our sample, this was the only correlate with the depression score. Even though the data were collected at a cross-sectional level and the temporal association between unemployment and a higher depression score is unclear, in periods of economic recession, having a job is perhaps one of the most important protective factors against depression.

None of the mental health subscales examined here were associated with HIV-related stigma or social support. Yet, on a univariable analysis, these subscales were associated with perceived access to medical care. Despite the fact that these associations became weaker after adjusting for other variables (depression maintained borderline significance), they still suggested that by reducing the psychological distress and improving the psychological well-being of the participants, the perception about accessibility to medical care improves [35]. 

Perceived access to care improved from baseline to the follow-up visit at TRIP. The change was minor and could be attributed, at least in part, to TRIP linking HIV-infected participants to care [24]. This is supported by the high score of perceived access to care among HIV-positive participants. Doing research together with taking action, such as linking HIV-infected participants to medical treatment in this intervention, may have enabled the participants to develop a better opinion about accessing medical care. Whitehead et al. commented on the importance of action research in health promotion [36]. Action research is a form of emancipatory research where the people involved in a situation study their social situation in order to improve their practice and their quality of understanding. Adopting action research methods in intervention programs such as TRIP and engaging participants in uncovering problems can facilitate the resolution of problems and improve their perception about HIV-related stigma and access to care. A better perceived access to medical care could result in improved health-seeking behavior. However, such behavior may be limited by the effective access to medical care. Thus, future research should focus not only on the perceived access to care, but also on the effective access, examining factors such as number of HIV tests and check-ups, antiretroviral treatment prescriptions, hospital admissions, etc.

There are certain limitations in this study. Firstly, most of the associations were examined cross-sectionally because mental health was only assessed at follow-up. Therefore, the temporal pattern between poor mental health and access to care could not be determined, thus their causal association could not be established. Secondly, depression, anxiety, and positive general affect were assessed using some items from the corresponding subscale of the Mental Health Inventory. As a result, comparisons with other studies were difficult. Due to the absence of some items from these scales, the use of cut-off points to screen for depression and anxiety was not possible. Since mental health indicators were examined as continuous variables, the interpretation of our findings as high scores could be less clinically relevant if they were not within the range of the case definition for depression or anxiety. Despite this, there are limited reports in the literature on the use of cut-offs for MHI-38 and its subscales [37], while there are references to the continuous scores [38,39]. Another limitation was that there was no diagnosis for mental health issues and no information was collected about history of psychiatric disorders or medication history. More widely used instruments with high sensitivity and specificity should be used in future studies to detect depression and anxiety in combination with clinical assessments. Lastly, the small sample size of some arms of this study may have reduced the effectiveness of the analysis and could have introduced a type II error, thus the lack of association should be interpreted with caution. Despite these limitations, the analysis provides an insight into the association of mental health with perceived access to care among participants of TRIP.

## 5. Conclusions

The only important risk factor for high depression scores among the participants of TRIP was being unemployed or unable to work, suggesting that, in periods of economic recession, working has a big impact on mental health. A better access to care score was noted among HIV-positive participants and among those with a lower depression score. Future interventions to reduce HIV transmission should also aim at improving the psychological well-being of participants, reducing their psychosocial distress, and minimizing perceived barriers to accessing medical care.

## Figures and Tables

**Table 1:** Socio-demographic characteristics of participants (*n* = 292) of the Transmission Reduction Intervention Project (TRIP) in Athens, Greece, 2013–2015.

Socio-Demographic Characteristics			Participant Group				
		Total	RS	NRS	LCS	NLCS	NC
Total		292	22 (7.5%)	136 (46.6%)	17 (5.8%)	47 (16.1%)	70 (24.0%)
Sex	Males	231 (79.1%)	17 (77.3%)	106 (77.9%)	14 (82.4%)	37 (78.7%)	57 (81.4%)
	Females	61 (20.9%)	5 (22.7%)	30 (22.1%)	3 (4.9%)	10 (21.3%)	13 (18.6%)
Age [years, median (interquartile range)]		35 (31–41)	39.5 (31–44)	35 (30–39)	36 (32–40)	34 (31–37)	36 (32–45)
Nationality	Greek	269 (92.1)	20 (90.9%)	123 (90.4%)	15 (88.2%)	42 (89.4%)	69 (98.6%)
	Non-Greek	23 (7.9%)	2 (9.1%)	13 (9.6%)	2 (11.8%)	5 (10.6%)	1 (1.4%)
Permanent residence	Locals (living in Athens since birth)	169 (57.9%)	12 (54.6%)	76 (55.9%)	10 (58.8%)	22 (46.8%)	49 (70%)
	Non-locals	123 (42.1%)	10 (45.4%)	60 (44.1%).	7 (41.2%)	25 (53.2%)	21 (30.0%)
Education	Up to high school	250 (85.6%)	19 (86.4%)	115 (84.6%)	15 (88.2%)	39 (83.0%)	62 (88.6%)
	Post-high school	42 (14.4%)	3 (13.6%)	21 (15.4%)	2 (11.8%)	8 (11.4%)	8 (11.4%)
Homelessness	Homeless	*39 (13.4%)*	*2 (9.1%)*	*24 (17.8%)*	*2 (11.8%)*	*9 (19.2%)*	*2 (2.9%)*
	Non-homeless	*252 (86.6%)*	*20 (90.9%)*	*111 (82.2%)*	*15 (88.2%)*	*38 (80.9%)*	*68 (97.1%)*
Employment	Unemployed/unable to work	232 (79.4%)	19 (86.4%)	113 (83.1%)	15 (88.2%)	35 (74.5%)	50 (71.4%)
	Employed	60 (20.6%)	3 (13.6%)	23 (16.9%)	2 (77.8%)	12 (25.5%)	20 (28.6%)
Injected drugs (past 6 months)	Injected drugs	*211 (72.3%)*	*18 (81.8%)*	*107 (78.7%)*	*13 (76.5%)*	*38 (80.9%)*	*35 (50.0%)*
	Non-injected drugs	*81 (27.7%)*	*4 (18.2%)*	*29 (21.3%)*	*4 (23.5%)*	*9 (19.1%)*	*35 (50.0%)*
Duration of drug injection [years, median (interquartile range)]		14 (8–18)	13.5 (4–19)	13 (7–18)	12 (7–16)	13.5 (8–15.5)	15 (9–21)
Drug/alcohol treatment at enrollment	On treatment	156 (53.4%)	15 (68.2%)	68 (50%)	12 (70.6%)	19 (40.4%)	42 (60.0%)
	Without treatment	136 (46.9%)	7 (31.8%)	68 (50%)	5 (29.4%)	28 (59.6%)	28 (40%)
Sexual orientation	Heterosexuals	284 (97.3%)	21 (95.5%)	133 (97.8%)	16 (94.1%)	46 (97.9%)	68 (97.1%)
	Non-heterosexuals	8 (2.7%)	1 (4.5%)	3 (2.2%)	1 (5.9%)	1 (2.1%)	2 (2.9%)

Numbers in italics stand for statistically significant differences among groups (*p* < 0.05). Abbreviations: RS, recent seeds; NRS, network of recent seeds; LCS, control seeds with long-term infection; NLCS, network of control seeds with long-term infection; NC, negative controls.

**Table 2:** Socio-demographic characteristics of participants of the Transmission Reduction Intervention Project (TRIP) based on human immunodeficiency virus (HIV) status.

Socio-Demographic Characteristics		HIV Status	
		Positive	Negative
Total		122	170
Sex	Males	94 (77.1%)	137 (80.6%)
	Females	28 (22.9%)	33 (19.4%)
Age [years, median (interquartile range)]		34 (30–40)	35 (32–41)
Nationality	Greek	108 (88.5%)	161 (94.7%)
	Non-Greek	14 (11.5%)	9 (5.3%)
Permanent residence	Local (living in Athens since birth)	64 (52.5%)	105 (61.8%)
	Non-locals	58 (47.5%)	65 (38.2%)
Education	Up to high school	107 (87.7%)	143 (84.1%)
	Post-high school	15 (12.3%)	27 (15.9%)
Homelessness	Homeless	*23 (19.0%)*	*16 (9.4%)*
	Non-homeless	*98 (81%)*	*154 (90.6%)*
Employment	Unemployed/unable to work	*106 (86.9%)*	*126 (74.1%)*
	Employed	*16 (13.1%)*	*44 (25.9%)*
Injected drugs (past 6 months)	Injected drugs	*108 (88.5%)*	*103 (60.6%)*
	Did not inject drugs	*14 (11.5%)*	*67 (39.4%)*
Duration of drug injection [years, median (Interquartile Range)]		13.5 (8–18)	14 (8–19)
Drug/alcohol treatment at enrollment	On treatment	72 (59.0%)	84 (49.4%)
	Without treatment	50 (41.0%)	86 (50.6%)
Sexual orientation	Heterosexuals	117 (95.9%)	167 (98.2%)
	Non-heterosexuals	5 (4.1%)	3 (1.8%)

Numbers in italics stand for statistically significant differences between groups.

**Table 3:** Mean scores and standard deviations for mental health subscales (self-reported). *p*-values for all comparisons among the study arms and between the HIV status groups were greater than 0.05.

Mental Health Subscales	Subscale Range		Participant Group [Mean, (sd)]					HIV Status [Mean, (sd)]	
		Overall	RS	NRS	LCS	NLCS	NC	Positive	Negative
Depression (*n* = 290)	4 to 23	14.1 (4.4)	12.8 (3.8)	13.9 (4.4)	13.6 (4.6)	14.6 (4.3)	14.5 (4.5)	13.9 (4.4)	14.2 (4.4)
Anxiety (*n* = 290)	9 to 54	32.5 (8.8)	31.9 (7.6)	31.7 (8.7)	29.4 (7.9)	33.9 (8.7)	34.0 (9.2)	31.9 (8.6)	32.9 (8.9)
General Positive Affect (*n* = 288)	10 to 60	28.6 (10.0)	30.2 (10.8)	29.4 (9.9)	30.8 (10.8)	26.3 (9.4)	27.8 (10.3)	28.7 (10.2)	28.6 (10.0)

Abbreviations: RS, recent seeds; NRS, network of recent seeds; LCS, control seeds with long-term infection; NLCS, network of control seeds with long-term infection; NC, negative controls.

**Table 4:** Univariable associations (A) between mental health subscale scores (self-reported) and socio-demographic characteristics, and (B) between mental health subscales scores (unadjusted β coefficients and 95% confidence intervals).

A		Mental Health Subscales		
Socio-Demographic Characteristics		Depression	Anxiety	General Positive Affect
Sex (males vs. females)		0.27 (−0.97, 1.52)	−1.62 (−4.11, 0.86)	−0.05 (−2.91, 2.80)
Age		0.02 (−0.04, 0.08)	−0.09 (−0.21, 0.04)	−0.01 (−0.16, 0.13)
Nationality (Greek vs. non-Greek)		0.42 (−1.49, 2.33)	1.99 (−1.84, 5.81)	1.47 (−3.02, 5.95)
Permanent residence (locals vs. non-locals)		0.09 (−0.94, 1.12)	1.15 (−0.90, 3.21)	0.69 (−1.67,3.06)
Education (up to high school vs. post-high school)		−0.56 (−2.00, 0.88)	−0.31 (−3.20, 2.57)	0.21 (−3.58, 3.17)
Homelessness (homeless vs. non-homeless)		0.87 (−0.63, 2.37)	−0.79 (−3.80, 2.22)	−3.03 (−6.46, 0.39)
Employment (unemployed/unable to work vs. employed)		*1.49 (0.24, 2.74)*	0.08 (−2.44, 2.60)	−1.29 (−4.18, 1.59)
Injected drugs (injected vs. did not inject drugs)(past 6 months)		0.75 (−0.38, 1.87)	0.64 (−1.62, 2.90)	−*3.21 (*−*5.77,* −*0.64)*
Duration of drug injection		0.06 (−0.00, 0.13)	0.04 (−0.09, 0.18)	−0.06 (−0.21, 0.09)
Drug/alcohol treatment at enrolment (on treatment vs. without treatment)		0.88 (−0.13, 1.89)	0.38 (−1.65, 2.41)	−1.25 (−3.59, 1.08)
Sexual orientation (heterosexuals vs. non-heterosexuals)		0.20 (−2.89, 3.29)	−3.50 (−9.68, 2.69)	−1.39 (−8.49, 5.70)
HIV status (HIV-positive vs. HIV-negative)		−0.29 (−1.32, 0.74)	−1.00 (−3.06, 1.05)	0.13 (−2.25, 2.50)
Participant group (vs. LCS)	RS	−0.87 (−3.66, 1.91)	2.50 (−3.05, 8.04)	−0.57 (−7.05, 5.92)
	NRS	0.30 (−1.92, 2.52)	2.24 (−2.18, 6.67)	−1.36 (−6.58, 3.86)
	NLCS	0.91 (−1.53, 3.35)	4.48 (−0.38, 9.34)	−4.40 (−10.13, 1.33)
	NC	0.84 (−1.49, 3.17)	4.59 (−0.06, 9.23)	−2.98 (−8.44, 2.49)
**B**				
**Mental Health Subscales**				
Depression		-	*1.38 (1.21*, *1.55)*	−*1.47 (*−*1.68*, −*1.27)*
Anxiety		-	-	−*0.55 (*−*0.67*, −*0.43)*
General positive affect		-	-	-

Numbers in italics stand for statistically significant relationships. Abbreviations: RS, recent seeds; NRS, network of recent seeds; LCS, control seeds with long-term infection; NLCS, network of control seeds with long-term infection; NC, negative controls; vs., versus.

**Table 5:** Univariable and multivariable regression models for perceived access to care.

			Crude β (95%CI)	Adjusted β (95%CI)
Socio-demographic characteristics	Sex (males vs. females)		−0.84 (−2.40, 0.72)	
	Age		−*0.08 (*−*0.16, 0.00)*	−0.05 (−0.12, 0.03)
	Nationality (Greek vs. non-Greek)		1.59 (−0.81, 3.99)	
	Permanent residence (locals vs. non-locals)		0.53 (−0.77, 1.84)	
	Education (up to high school vs. post-high school)		0.40 (−1.40, 2.20)	
	Homelessness (homeless vs. non-homeless)		−1.41 (−2.44, 1.13)	
	Employment (unemployed/unable to work vs employed)		−0.62 (−2.23,0.99)	
	Injected drugs (injected vs. did not inject drugs—past 6 months)		−0.27 (−1.70, 1.16)	
	Duration of drug injection		−0.02 (−0.11, 0.06)	
	Drug/alcohol treatment at enrolment (on treatment vs. without treatment)		0.80 (−0.49, 2.09)	
	Sexual orientation (heterosexuals vs. non-heterosexual)		−2.00 (−5.80, 1.80)	
	HIV status (HIV-positive vs. HIV-negative)		*1.49 (0.19, 2.79)*	*1.32 (0.11, 2.54)*
	Participant group (vs. LCS)	RS	1.19 (−2.39, 4.77)	
		NRS	−0.04 (−2.93, 2.85)	
		NLCS	1.57 (−1.63, 4.76)	
		NC	−0.33 (−3.36, 2.70)	
Measures	Mental health (self-reported)	Depression	−*0.29 (*−*0.44,* −*0.14)*	−*0.22 (*−*0.43, 0.00)*
		Anxiety	−*0.14 (*−*0.21,* −*0.06)*	−0.06 (−0.16, 0.03)
		General positive affect	*0.08 (0.01, 0.14)*	0.49 (−0.10, 0.05)
	Stigma and support	HIV-related stigma	−0.47 (−1.33, 0.38)	
		HIV-related social support	0.73 (−0.00, 1.47)	
	Perceived access to care at baseline		*0.34 (0.23, 0.45)*	*0.33 (0.22, 0.43)*

Numbers in italics stand for statistically significant relationships. Abbreviations: RS, recent seeds; NRS, network of recent seeds; LCS, control seeds with long-term infection; NLCS, network of control seeds with long-term infection; NC, negative controls; vs., versus.

## Data Availability

The data presented in this study may be available on request from the corresponding author.

## Supplementary Materials

The following are available online at https://www.mdpi.com/2077-0383/10/6/1181/s1, Table S1: Questions (Q) and responses (R) of TRIP questionnaire related to mental health, stigma and social support experiences, and perceived access to care.
