# Supplementary material for: Mental Health and Perceived Access to Care among People Who Inject Drugs in Athens, Greece
Source: J Clin Med. 2021 Mar 12;10(6):1181. doi: 10.3390/jcm10061181 (PMC8002050; doi:10.3390/jcm10061181)
Supplement: Supplementary file 1 [file jcm-10-01181-s001.pdf]

Table S1. Questions (Q) and responses (R) of TRIP (Transmission Reduction Intervention Project) questionnaire related to mental health, stigma and social support experiences, and perceived access to care.

**Psychological distress**	
	**Depression**
Q1	Have you felt downhearted and blue?
Q2	Have you been moody or brooded about things?
Q3	Have you been in low or very low spirits?
R1-R3	(1) None of the time (2) A little of the time (3) Some of the time (4) A good bit of the time (5) Most of the time (6) All of the time
Q4	Did you feel depressed during the past month?
R4	(1) No, never felt depressed at all<br>(2) Yes, a little depressed now and then<br>(3) Yes, quite depressed several times<br>(4) Yes, very depressed almost every day<br>(5) Yes, to the point that I did not care about anything for days at a time
	**Anxiety**
Q5	Have you been a very nervous person?
Q6	Have you felt tense or "high-strung"?
Q7	Have you felt restless, fidgety, or impatient?
Q8	How often did you become nervous or jumpy when faced with excitement or unexpected situations during the past month?
Q9	During the past month, how often did your hands shake when you tried to do something?
Q10	During the past month, how often did you get rattled, upset or flustered?
Q11	How often during the past month did you find yourself trying to calm down?
Q12	How much have you been bothered by nervousness, or your "nerves", during the past month?
Q13	During the past month, have you been anxious or worried?
R5-R13	(1) None of the time (2) A little of the time (3) Some of the time (4) A good bit of the time (5) Most of the time (6) All of the time
**Psychological well-being**	
	**General positive affect**
Q14	Have you felt that the future looks hopeful and promising?
Q15	Has your daily life been full of things that were interesting to you?
Q16	Did you feel relaxed and free from tension?
Q17	Have you generally enjoyed the things you do?
Q18	Have you felt calm and peaceful?
Q19	Has living been a wonderful adventure for you?
Q20	Have you felt cheerful, lighthearted?
Q21	Were you a happy person?

Q22	When you have got up in the morning, this past month, about how often did you expect to have an interesting day?
Q23	How often, during the past month, have you been waking up feeling fresh and rested?
R14-R23	(1) None of the time (2) A little of the time (3) Some of the time (4) A good bit of the time (5) Most of the time (6) All of the time
**Stigma related to HIV**	
Q24	In the last 6 months, has anyone made nasty comments to you because they thought you had recent HIV infection?
Q25	In the last 6 months, has anyone threatened or physically attacked you because they thought you had recent HIV infection?
Q26	In the last 6 months, has anyone denied you access to goods or forbidden you to go somewhere because they thought you had recent HIV infection?
Q27	In the last 6 months, has anyone excluded you from social gatherings or have you gotten invited to fewer social events because they thought you had recent HIV infection?
R24-R27	(0) No (1) Yes
**Social support for HIV**	
Q28	In the last 6 months, has anyone offered you emotional support because they thought you had recent HIV infection?
Q29	In the last 6 months, has anyone offered you any form of concrete assistance like money or food because they thought you had recent HIV infection?
Q30	In the last 6 months, has anyone offered you information about where you can get any HIV service /testing/ consultation, etc. because they thought you had recent HIV infection?
R28-R30	(0) No (1) Yes
**Access to care**	
Q31	I am able to get medical care whenever I need it.
Q32	It is hard for me to get medical care in an emergency.
Q33	Sometimes I go without the medical care I need because it is too expensive.
Q34	I have easy access to the medical specialists that I need.
Q35	Places where I can get medical care are very conveniently located.
R31-R35	(0) Strongly disagree (1) Somewhat disagree (2) Neither agree nor disagree (3) Somewhat agree (4) Strongly agree
